# Preventive and curative effect of difenoconazole + azoxytrobin and thiophanate-methyl against lucky bamboo anthracnose disease caused by *Colletotrichumdracaenophilum*

**DOI:** 10.1016/j.heliyon.2023.e14444

**Published:** 2023-03-11

**Authors:** Ibrahim E. Elshahawy, Osama M. Darwesh

**Affiliations:** a Plant Pathology Department, National Research Centre, Cairo, 12622, Egypt; b Agricultural Microbiology Department, National Research Centre, Cairo, 12622, Egypt

**Keywords:** Lucky bamboo, Anthracnose, Thiophanate-methyl, Difenoconazole + azoxytrobin

## Abstract

In Egypt, *Dracaena sanderiana* (lucky bamboo) is an ornamental plant imported from several countries. Two weeks after they arrived at the nurseries, anthracnose indications were detected on the shoots of imported *D. sanderiana* samples. Four *Colletotrichum* spp. isolates were obtained from the symptomatic lucky bamboo plants. The obtained isolates belonged to the species of *C. gloeosporioides* or *C. dracaenophilum* based on their morphological characteristics and molecular biology analyses. Pathogenicity tests reveal that *C. dracaenophilum* isolate 4 was found to be more pathogenic than the other isolates. The *in vitro* investigation was conducted with the objectives of evaluating six systemic fungicides for their inhibitory effect against *C. dracaenophilum*. Data reveal that, thiophanate-methyl and difenoconazole + azoxytrobin at ≥15 ppm completely inhibited the pathogen growth. Tebuconazole and flusllazole inhibited growth completely at ≥20 ppm, whereas iprodione and cyprodinil + fludioxonil had a lower effect (56.6 and 54.4% reduction, respectively) at this dose. The *in vivo* investigation was conducted with the objectives of evaluating the preventive and curative effects of the most effective fungicides against anthracnose disease. Lucky bamboo plants were treated with fungicide and either inoculated or not with *C. dracaenophilum* before being left for 25 or 60 days. On both insidiously infected and vaccinated lucky bamboo plants, the combination of difenoconazole, azoxytrobin, and thiophanate-methyl at 20 ppm greatly reduced the development of anthracnose. Tebuconazole and flusllazole were found to be phytotoxic.

## 1 Introduction

Lucky bamboo (*Dracaena sanderiana* = *D. braunii* hort. Sander ex Mast.) is an *Asparagaceae* family evergreen perennial ornamental plant native to Cameroon in West Africa. It’s raised as a decorative foliage vase plant and for low-light interiorscapes [1]. Some of the popular names for lucky bamboos include friendship bamboo, the golden bamboo, ribbon plant, Belgian evergreen, belly bamboo, curly bamboo, Chinese water bamboo, pot bound, and bamboos. Lucky bamboo is a valuable commodity in our lives because it is used to decorate both private and public spaces, such as homes, offices, schools, and shopping malls [2,3]. In Egypt, it has recently gained popularity as a decorative houseplant due to its attractive appearance, low cost, ability to grow in a variety of indoor conditions, and lack of experience required to care for it [4,5]. Several diseases affect *Dracaena* species. Anthracnose, Botrytis blight, stem and leaf spots, and Fusarium stem rot are examples of fungal diseases, while Erwinia leaf rot and stem spot are examples of bacterial diseases [[6], [7], [8]]. However, lucky bamboo production is severely hampered by the anthracnose disease, which is brought on by *Colletotrichum* species, causing enormous economic losses throughout the world [4,[9], [10], [11]]. *Colletotrichum* is a large genus of *Ascomycete* fungi that contains species that cause anthracnose diseases on a variety of economically important crops [12]. Anthracnose is a term used to describe plant diseases characterized by dark, sunken lesions containing spores [13].

**Fig. 1:**
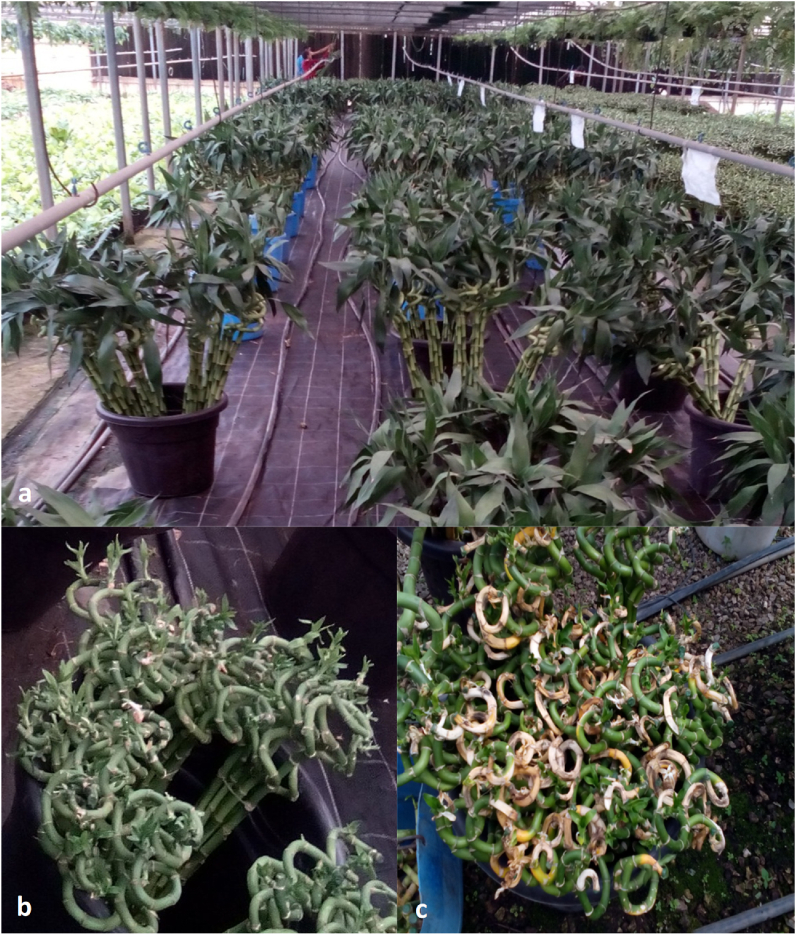
Healthy and diseased lucky bamboo plants observed in Flora mix-Egypt Company. (a) Lucky bamboo plants (general view of the nursery). (b) Healthy and (c) infected (with typical anthracnose symptoms) lucky bamboo plants (2 weeks after arrives to the nursery).

*D. sanderiana* plants with typical anthracnose symptoms were observed in Flora mix-Egypt Company in spring 2021. Flora mix-Egypt is a leading grower, importer and exporter of a diverse range of premium cut flowers and indoor plants, Imbaba, Giza, Egypt. The infected plants had beige lesions on their stalks with a dark brown border that ultimately turned necrotic and covered the entire stalk (Fig. 1a). There were several black fruiting bodies (acervuli) within the lesions, along with characteristic *Colletotrichum* conidia and setae, and the plants' leaves had wilted and fallen off (Fig. 1b and c). The fact that healthy-looking plants began to exhibit symptoms roughly two weeks after they were delivered to the retailers, according to store personnel, suggests that the plants were already infested but asymptomatic when they reached in Egypt. Though *C. dracaenophilum* has been isolated from a lucky bamboo plant in Egypt later [4]. The pathogenicity of the isolated isolate was also established in accordance with Koch’s postulate. However, morphological study data by themselves are insufficient. As a result, molecular methods are required to make up for morphological characterization’s shortcomings. On the other hand, because this pathogen can attack a number of plant hosts, other members of the *C gloeosporioides* species complex, in addition to *C. dracaenophilum*, may be able to infect lucky bamboo [9].

Chemical protection is a primary strategy for plant disease control. In the absence of a resistant source, the most common way to protect plants from diseases is to use fungicides. According to Morsy and Elshahawy [4] and Abdel-Rahman et al. [5], the systemic fungicide carbendazim is effective *in vitro* against *Colletotrichum* spp. Thiophanate methyl, difenoconazole + azoxytrobin, iprodione, tebuconazole, flusllazole, and cyprodinil + fludioxonil have all shown promise in the control of *Colletotrichum* in other crops [11,[14], [15], [16]]. As a result, the following objectives were pursued: 1) isolate, characterize and identify the *Colletotrichum* spp. associated with anthracnose of lucky bamboo (*D. sanderiana*) in Egypt; 2) determine variation in aggressiveness and achieve Koch’s postulates with the obtained isolates; 3) determine *in vitro* sensitivity of the most aggressive isolate to a few commonly used fungicides; and 4) Test fungicides for *Colletotrichum* control in inoculated plants and asymptomatic but infected rooted cuttings.

## 2 Material and methods

### 2.1 Isolation and morphological identification of *Colletotrichum* isolates

One hundred plant samples of lucky bamboo, *D. sanderiana* with anthracnose infection were collected from the commercial nursery of Flora mix-Egypt, a large importer and exporter of ornamental plants located in Giza governorate, Egypt (Fig. 1a). Plant samples were divided to 10 groups, each group contained 10 plants. Isolations were created from affected stalks with anthracnose lesions. Diseased stalks with lesions and acervuli were cut into small pieces (1 cm) with a sterile scalpel and surface sterilized with sodium hypochlorite at 1.0% (v/v) in sterile water for 1 min, rinsed three times with sterile distilled water, and air-dried on sterile filter paper for 30 min inside the laminar flow. Surface-sterilized tissues were placed on Petri plates containing potato dextrose agar (PDA, Difco Laboratories®, Detroit, MI, USA), acidified with lactic acid (2.5 mL of 25% [v/v] per liter of medium) to minimize bacterial growth (APDA), and incubated at room temperature (25 ± 2 °C). After 7 days incubation, monoconidial cultures were inoculated onto water agar medium (WA). When a single conidium germinated, it was transferred to APDA using a sterilized needle. The description of Mordue [17] and the key of Sutton [18] were used as references to identify the fungi at the genus level. Four *Colletotrichum* species isolates were obtained based on morphological identification. All of the isolated single-conidium isolates were used for further characterization and pathogenicity testing. The frequencies of isolated isolates were calculated based on the colony’s morphology as follows: Frequency of isolate (%) = number of isolate colonies/Total number of colonies × 100.

### 2.2 Molecular identification

The internal transcribed spacer (ITS) region of ribosomal DNA (rDNA) from the isolated fungi was sequenced to confirm the initial morphological identification. *Colletotrichum* spp. isolates were sub-cultured on potato dextrose broth medium (PDB, Difco Laboratories®, Detroit, MI, USA). Each isolate was inoculated in 50 mL of PDB and incubated for 10 days in a shaker incubator at 25 ± 2 °C with 120 rpm. After incubation period, the 4 isolates' fungal mycelium was harvested. The CTAB protocol was used to extract DNA from mycelium [19]. The DNA from the various isolates was amplified using polymerase chain reaction (PCR) with the ITS1 and ITS4 primer sets. To amplify the ITS region of the 18S rDNA gene, the primers ITS1: (5′TCCGTAGGTGAACCTGCGG-3′) and ITS4: (5′TCCTCCGCTTATTGATATGC-3′) were used (partial sequence). 1 × PCR buffer (NEB, England), 1 nmol of dNTPs, 1 pmol of 2 mM MgSO_4_, 0.25 pmol of forward and reverse primers, 1 unit Taq DNA polymerase (NEB, England), and 10 μl template DNA were included in the final 50 μl reaction mixture. The PCR program began with a 2 min initial denaturation at 94 °C, followed by 35 cycles of denaturation at 95 °C for 30 s, annealing at 56 °C for 30 s, elongation at 72 °C for 60 s, and final extension at 72 °C for 10 min. The amplified products were separated on 1% agarose gels with a 1× TBE (Tris-borate-EDTA) buffer. UV light was used to photograph the gels. The PCR products were purified using the QIAquick Gel Extraction Kit (QIAGEN, USA) and run on an agarose gel to obtain 18S rDNA fragments for sequencing. The identification was accomplished by comparing the contiguous 18S rDNA sequence to the 18S rDNA sequence data from the reference and type strains available in the public database GenBank using the BLAST program (National Center for Biotechnology Information, https://blast.ncbi.nlm.nih.gov/Blast.cgi). The Jukes Cantor Model was used to align the sequences. The sequences of these strains were entered into GenBank and assigned accession numbers. The evolutionary distance was calculated using the parameter model, and the phylogenetic tree was built using the neighbor-joining method [20,21].

### 2.3 Pathogenicity tests

Pathogenicity tests were performed on one week-old (after arriving at the stores) bare-rooted, healthy-looking bamboo plants (70 cm long). These plants were kept at room temperature for two months to eliminate latent infections. Following that, pathogenicity procedures were carried out. Four plants were planted in glass jars with wide mouths and a capacity of 2 L, each containing 1 L of chlorine free sterilized (with filtration) water. The stalks were submerged in water for about 20 cm. By severing the top of the stem with a sterile blade and inserting a mycelial agar plug, Lucky bamboo seedlings were inoculated with 10-day-old single-spore cultures cultured on acidified PDA for each isolate (5 mm in diameter). The negative control was a comparable plug of sterile acidified PDA agar. Parafilm strips were then used to cover the inserted plugs [22]. Each isolate received ten replicates in the randomized complete block design trials (jars). The inoculated and control plants were kept at 25 ± 5 °C (room temperature). Water in the jars was changed with chlorine free sterilized (with filtration) water every seven days. Daily symptom development checks were performed on the plants, and disease severity was calculated 25 days after inoculation. Anthracnose disease severity (DS) in inoculated lucky bamboo plants was assessed 25 days after inoculation using a 0–5 rating suggested by Moral et al. [23], with some modifications as follows: 0 = No signs of disease, 1 = Yellowing lesions with or without acervuli that cover approximately 1–10 cm of the stalk, 2 = Yellowing lesions with or without acervuli covering approximately 11–20 cm of the stalk, 3 = Yellowing lesions with or without acervuli covering approximately 21–30 cm of the stalk, 4 = Yellowing lesions with or without acervuli covering approximately 31–40 cm of the stalk, and 5 = Yellowing lesions with or without acervuli covering more than 41 cm of the stalk. In each replication, a disease severity index (DSI) was calculated using the following formula: DSI = [(Ʃni × i)/(N × 5)] × 100, where i represents severity (from 0 to 5), ni is the number of stalks with severity, N is the total number of stalks, and 5 is the highest value of severity. Following the final evaluations, Lesions and acervuli on stem tissues were disinfected with a 1.0% sodium hypochlorite solution and then rinsed in deionized sterile water. Symptomatic tissue was aseptically excised and then placed to acidified PDA plates. Under a light microscope, the hyphal growth, conidial shape and size of the plates were evaluated for the occurrence of *Colletotrichum* after a 7-day incubation period at 25 ± 5 °C.

### 2.4 *In vitro* sensitive of *C. dracaenophilum* to chemical fungicides

Six systemic fungicides were chosen and used based on their ability to control anthracnose disease in other crops. To preserve the activity of these fungicides, commercial formulations were purchased from local retailers and stored in desiccators at 25 °C (Table 1). *C. dracaenophilum* isolate No. 4 (the most virulent isolate) was tested for fungicide sensitivity using modified agar assays [24,25]. Molten acidified PDA was amended with the fungicide formulation in this assay to achieve final desired concentrations of 5.0, 10.0, 15.0, and 20.0 ppm for each fungicide. The above concentrations were converted to g a.i./L based on the active ingredient of each fungicide before being added to potato dextrose agar (PDA) medium in petri dishes. Agar plugs (5 mm in diameter) were removed from the advancing edge of a *C. dracaenophilum* colony, grown on PDA medium for 6 days, and placed centrally, mycelia-side down, on the surface of the PDA dishes. Dishes were incubated in the dark for 7 days at 25 ± 2 °C. Agar plugs inoculated on fungicide-free PDA dishes served as controls. Five times, the procedures were repeated. Colony growth (mm) was measured 7 days after incubation, and the mean of two colony diameters taken at right angles was computed. Using Vincent’s equation, the percentage inhibition (PI) of mycelial growth was obtained [26], PI = 100(C − T)/C (where C = diameter of control mycelial growth; T = diameter of treated mycelial growth). The mean values of the previously arcsine transformed PI values were examined using an ANOVA of a factorial design (6 × 1 × 4). The statistical study was performed using the CoStat6303Win.exe program. The significance of interventions was assessed by the Duncan’s multiple range test (P = 0.05).

**Table 1:** Fungicides used for *in vitro* amended agar assays.

Trade name	Active ingredient	Formulation	Manufacturer
**Actamyl**	70%Thiophanate-methyl	WP	Arrest Life Science Company - France
**Amstar Top**	12.5% Difenoconazole + 20% Azoxytrobin	EC	Syngenta Agro Switzerland
**Chipico**	50% Iprodione	WP	Jiangsu Kuaida Agrochemical Co Ltd- China
**Folicure**	25% Tebuconazole (triazole)	EW	Bayer CropScience Ltd. United Kingdom
**Only one**	40% Flusllazole	EC	Tianjin Shengxinhai Chemical Co., Ltd. China
**Switch**	37.5% w/w Cyprodinil + 25% w/w Fludioxonil	WG	Syngenta Agro Switzerland

### 2.5 Preventive effect of chemical fungicides

Based on *in vitro* data on bamboo, four systemic fungicides were examined for prevention and treatment of anthracnose disease: Actamyle (Thiophanate-methyl 70%, 0.028 g a.i./L of water = 20 ppm); Amstar Top (Azoxystrobin 20% + Difenoconazole 12.5%, 0.062 g a.i./L of water = 20 ppm); Folicure (Tebuconazole (triazole) 25%, 0.08 g a.i./L of water = 20 ppm) and Only one (Flusllazole 40%, 0.05 g a.i./L of water = 20 ppm). To achieve preventive effects, one week-old rooted cuttings of lucky bamboo plants were kept for two months at room temperature to eliminate latent infections before being sprayed with a hand sprayer until runoff and kept in wide-mouthed, 2 L capacity glass jars containing 1 L water until inoculation. These jars were also amended with the same fungicide concentration as before. Water in the jars was changed with chlorine free sterilized (with filtration) water every seven days. The fungicide treatment was repeated at 7-day intervals, so each group received three treatments. To prevent contamination, control plants were similarly sprayed with sterile water and stored in separate glass jars. Twenty four hours after fungicide spraying, the plants were inoculated by a mycelia agar plug (5 mm in diameter) into a cut on the top half of the stalk while being maintained at room temperature as previously explained. There were ten treatments: 1) Control plants, inoculated with a sterile agar plug; 2) Inoculated plants, inoculated with *C. dracaenophilum*; 3) Control + thiophanate-methyl; 4) Control + azoxystrobin + difenoconazole; 5) Control + tebuconazole; 6) Control + flusllazole; 7) Inoculated + thiophanate-methyl; 8) Inoculated + azoxystrobin + difenoconazole; 9) Inoculated + tebuconazole; 10) Inoculated + flusllazole. The experiment used a randomized complete block design with ten replications (jars) for each treatment. Plants were checked daily for symptom development, and disease severity (DS) was measured on each plant 25 days after inoculation using a 0–5 rating suggested by Moral et al. [23], with some modifications as previously mentioned in the pathogenicity test.

### 2.6 Curative effect of chemical fungicides

Based on preventive effect data, Folicure and flusllazole fungicides were found to be phytotoxic and were removed from this experiment. Therefore, two systemic fungicides were examined for curative effect against anthracnose disease: Actamyle (Thiophanate-methyl 70%, 0.028 g a.i./L of water = 20 ppm) and Amstar Top (Azoxystrobin 20% + Difenoconazole 12.5%, 0.062 g a.i./L of water = 20 ppm). One-week-old rooted cuttings of lucky bamboo plants were sprayed with each fungicide, but they weren’t inoculated, and left in the same conditions for two months to see if the fungicides had any curative effects on *Colletotrichum* spp. This was done to see if disease would spread from asymptomatic plants and if the fungicides had any effect on the species. Lucky bamboo plants were sprayed with each fungicide with a hand sprayer until runoff, then placed in wide-mouthed, 2 L glass jars containing 1 L water. These jars were also amended with the same fungicide concentration as before. Water in the jars was changed with chlorine free sterilized (with filtration) water every seven days. The fungicide treatment was repeated at 7-day intervals, so each group received three treatments. There were three treatments: 1) Untreated plants; 2) Thiophanate-methyl and 3) Azoxystrobin + difluconazole. The experiment used a randomized complete block design with ten replications (jars) for each treatment. Plants were checked daily for symptom development, and the incidence of anthracnose in each group was measured 60 days after treatment.

### 2.7 Statistical analysis

Before statistical analysis, the data were checked for normality and variance uniformity. An arcsine square root transformation was used to change percentage data in order to improve variance homogeneity, but untransformed data were also displayed. All data were subjected to an analysis of variance to see if there was a significant difference between the means (ANOVA). The Duncan’s multiple range test was estimated to compare the means at P = 0.05 using the CoStat6303Win.exe program [27].

## 3 Results

### 3.1 Isolation and identification of *Colletotrichum* isolates

**Fig. 2:**
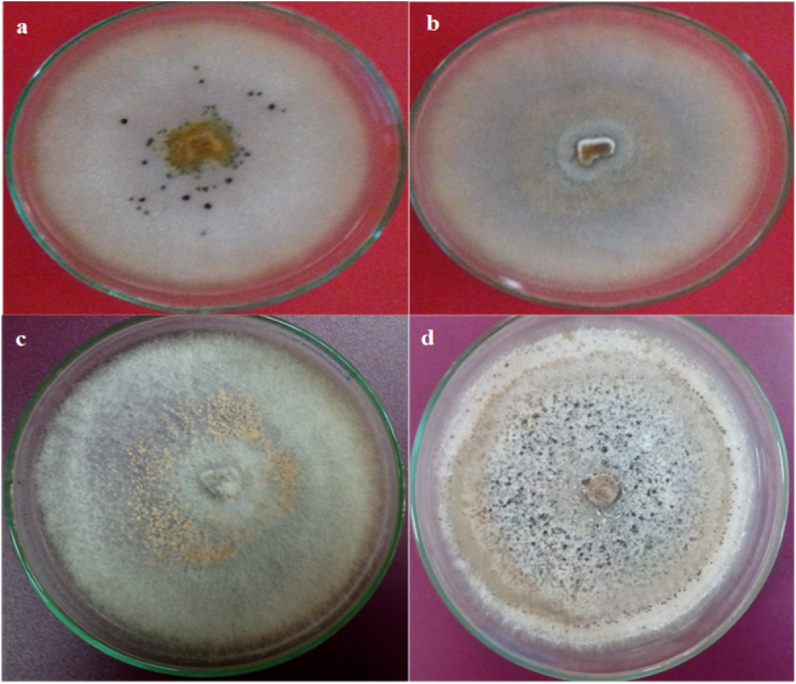
Colonies of *Colletotrichum* isolates, isolated from diseased lucky bamboo stems showing symptoms of anthracnose disease, 7 days old on PDA. (a) isolate 1 (*C. dracaenophilum*), (b) isolate 2 (*C. gloeosporioides*), (c) isolate 3 (*C. gloeosporioides*) and (d) isolate 4 (*C. dracaenophilum*).

**Fig. 3:**
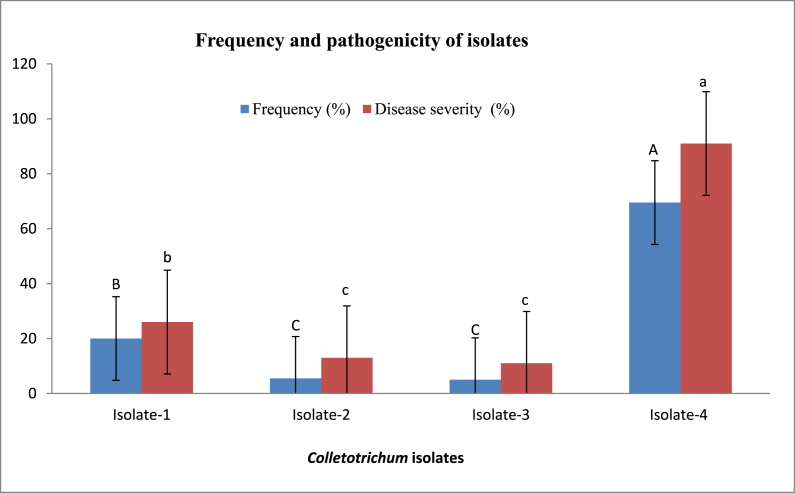
Frequency and pathogenicity of the obtained *Colletotrichum* isolates. Values are means of ten replications. Frequency of isolate (%) = number of isolate colonies/Total number of colonies × 100. Pathogenicity tests were performed by inserting a mycelial agar plug (5 mm in diameter) into a cut (with a sterile blade) on the upper half of the stalk. At 25 days after inoculation, anthracnose disease severity (DS) in inoculated lucky bamboo plants was evaluated using a 0–5 rating suggested by Moral et al. [23]. In each replication, a disease severity index (DSI) was computed using the following formula: DSI = [(ni × i)/(N × 5)] × 100, where ni is the total number of stalks, N is the number of severe stalks, and i indicates a severity (zero to five). According to Duncan’s multiple range tests, bars within each variable with the same letter show that the means and standard errors are not significantly different at P = 0.05. For analyses of variance, percentage data were converted using the arcsine square-root transformation; however, untransformed data are shown here.

**Fig. 4:**
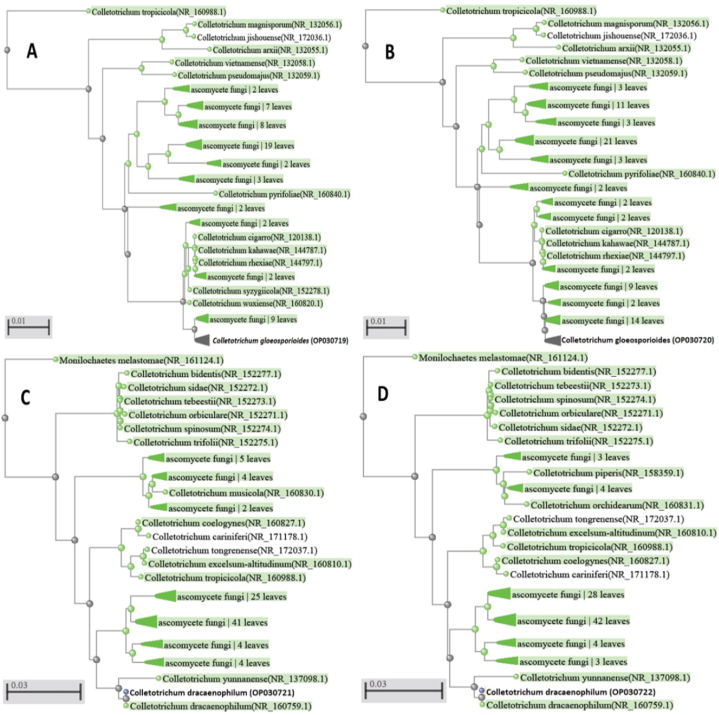
The phylogenic tree showed convergence between our isolates and Genebank closed strains. Isolates (A and B) were matched with those for the *C. gloeosporioides* species complex. Isolates (C and D) were putatively considered to be *C. dracaenophilum*.

From symptomatic lucky bamboo stems, four *Colletotrichum* isolates were isolated. The two isolates (2 and 3) were matched with the *C. gloeosporioides* species complex (Penz.) Penz. & Sacc (Fig. 2b and c). Colonies of isolates 1 and 4 on acidified PDA started off white before turning somewhat pink in the center of each colony (Fig. 2a and d). These colonies appeared to be *C. dracaenophilum* based on their colour and appearance as well as microscopic analysis of the conidia and setae. The data in Fig. (3), revealed significant differences in the frequency of the four isolates, with *C. dracaenophilum* (isolate 4) having the highest frequency (69.5%). Other isolates had a low frequency of 20.0, 5.5 and 5.0% for isolates 1, 2, and 3, respectively. To verify the first morphological identification, the ribosomal DNA (rDNA) from the isolated fungus' internal transcribed spacer (ITS) region was sequenced. Using the BLAST tool, the resulting sequencing data were compared to the National Center for Biotechnology Information’s global registered database (NCBI). The phylogenetic trees revealed that these isolates were very similar to the type isolates of *C. gloeosporioides* species complex (isolates 2 and 3) and *C. dracaenophilum* (isolates 1 and 4) that were placed in the NCBI Culture Collection Center and uploaded to GenBank under the accession numbers OP030719, OP030720, OP030721, and OP030722 (Fig. 4A–D).

### 3.2 Pathogenicity tests

**Fig. 5:**
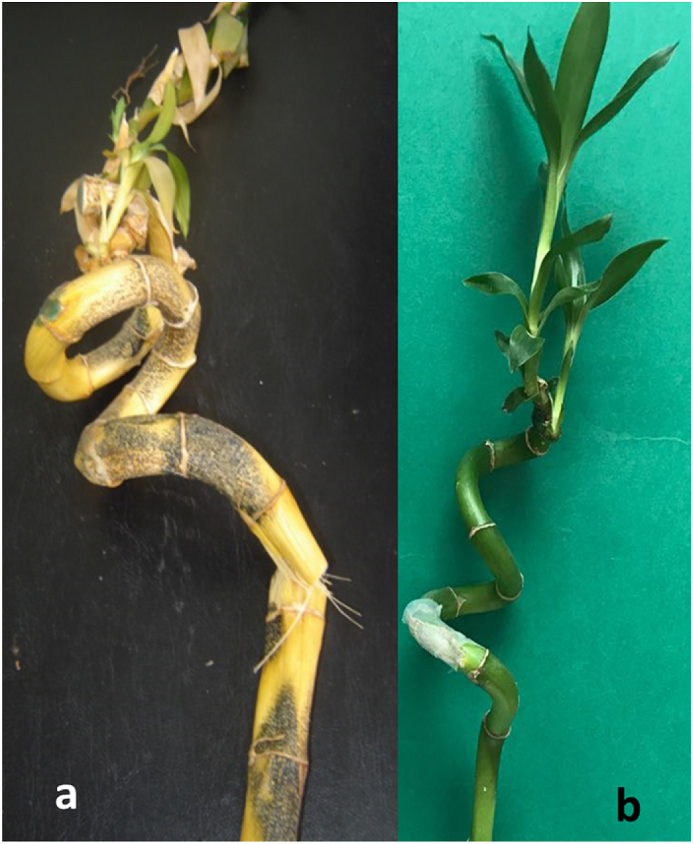
Pathogenicity test of isolate 4 (*C. dracaenophilum*) on lucky bamboo stems. (a), typical symptoms of anthracnose disease (numerous black fruiting bodies (acervuli) visible within the lesions and spread to the entire stalk) induced by *C. dracaenophilum* isolate 4. (b), control (lucky bamboo stem inoculated with plug of sterile acidified PDA agar free from *C. dracaenophilum*). Photographs were taken 25 days after inoculation.

Four healthy lucky bamboo plants were grown in a 2.0 L glass jar filled with filtered sterilized water to evaluate the pathogenicity of the recovered *Colletotrichum* isolates in order to support Koch’s postulates. Mycelium plugs (0.5 cm in diameter) were cut from a 7-day old colony of each isolate grown on PDA medium and directly inoculated into lucky bamboo plants. The remaining four lucky bamboo plants, which served as controls, were inoculated with the same size plugs of un-inoculated PDA. All of the plants were incubated at room temperature (25±5 °C). After 25 days, all of the lucky bamboo plants that received the *Colletotrichum* spp. inoculation displayed classic anthracnose signs, but the control plants showed no symptoms throughout the whole assessment time (25 days) (Fig. 3). Disease symptoms included light yellow lesions that spread from the inoculated area of the stem. Ten days after inoculation, the dark brown acervuli initially appeared and expanded throughout the entire fortunate bamboo stem (Fig. 5a and b). Anthracnose symptoms similar to those seen in nurseries appeared on *C. dracaenophilum* isolate 4 and isolate 1 inoculated plants, with disease severity of 91 and 26.0%, respectively (Fig. 3). Plants inoculated with isolate 2 and 3 developed diseases in a similar pattern, with disease severity of 13.0, and 11.0%, respectively (Fig. 3). The fungal pathogen was re-isolated from symptomatic plant tissues and displayed the same morphological features as the inoculated isolate. In order to conduct additional testing, *C. dracaenophilum* isolate 4 was chosen based on pathogenicity data.

### 3.3 *In vitro* inhibitory effect of chemical fungicides toward *C. dracaenophilum*

For fungicides and concentrations, as well as their interaction with *C. dracaenophilum* mycelial development, the ANOVA was extremely significant (P = 0.05) (Table 2). After 7 days of incubation, colony growth of *C. dracaenophilum* was completely inhibited at concentration of 15 ppm thiophanate-methyl and difenoconazole + azoxytrobin. At 15 ppm, the average inhibition was 88% for tebuconazole and 84.6% for flusllazole (*P* = 0.05). At this concentration, the remaining fungicides had less of an effect (*P* = 0.05). After 7 days of incubation, colony growth of *C. dracaenophilum* was completely inhibited with tebuconazole and flusllazole at concentrations of ≥20 ppm. On average, thiophanate-methyl, difenoconazole + azoxytrobin, tebuconazole, and flusllazole treatments were the most effective, while iprodione and cyprodinil + fludioxonil treatments were the least effective (Table 2).

**Table 2:** Inhibition (%) of colony growth of *C. dracaenophilum* isolate 4 by different fungicides using *in vitro* amended agar assays.

Fungicide	Concentration (ppm)				
	5	10	15	20	Average
Thiophanate-methyl	45.8 i	73.2 e	100.0 a	100.0 a	79.8 AB
Difenoconazole + Azoxytrobin	74.2 e	94.4 b	100.0 a	100.0 a	92.2 A
Iprodione	06.6 n	24.0 l	36.4 j	56.6 g	30.9 C
Tebuconazole	36.0 j	65.8 f	88.0 c	100.0 a	72.5 B
Flusllazole	27.6 k	53.0 h	84.6 d	100.0 a	66.3 B
Cyprodinil + Fludioxonil	09.8 m	25.8 kl	37.4 j	54.4 gh	31.9 C

Observations made 7 days after inoculation when the control colony covered petri plate (0% inhibition). Each value is the mean of four replicates. Multiple comparisons of means are based on arcsin-transformed values. However, mean percentages are shown. In each column or row values for the combined fungicide × concentration treatments followed by a different letter are significantly different according to Duncan’s multiple range test at *P* = 0.05.

### 3.4 Preventive effect of thiophanate-methyl and difenoconazole + azoxytrobin

**Fig. 6:**
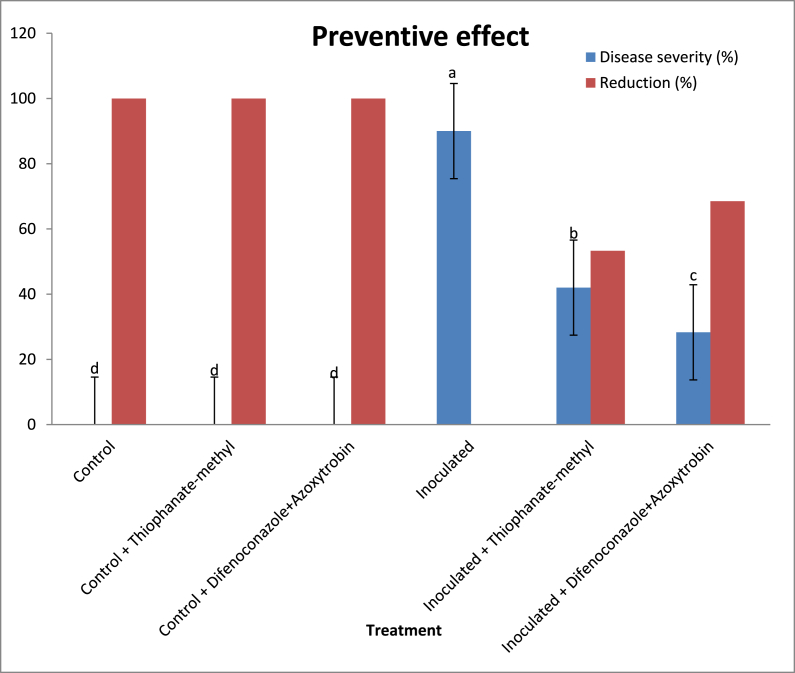
Preventive effect of thiophanate-methyl and difenoconazole + azoxytrobin against anthracnose disease severity (%) of lucky bamboo plants inoculated with *C. dracaenophilum*, 25 days after inoculation. Values are means of ten replications. One week-old rooted cuttings of lucky bamboo plants were kept for two months at room temperature to eliminate latent infections before being treated with each fungicide and pathogen inoculation. At 25 days after inoculation, anthracnose disease severity (DS) in inoculated lucky bamboo plants was evaluated using a 0–5 rating suggested by Moral et al. [23]. According to Duncan’s multiple range tests, bars within each variable with the same letter show that the means and standard errors are not significantly different at P = 0.05. For analyses of variance, percentage data were converted using the arcsine square-root transformation; however, untransformed data are shown here.

**Fig. 7:**
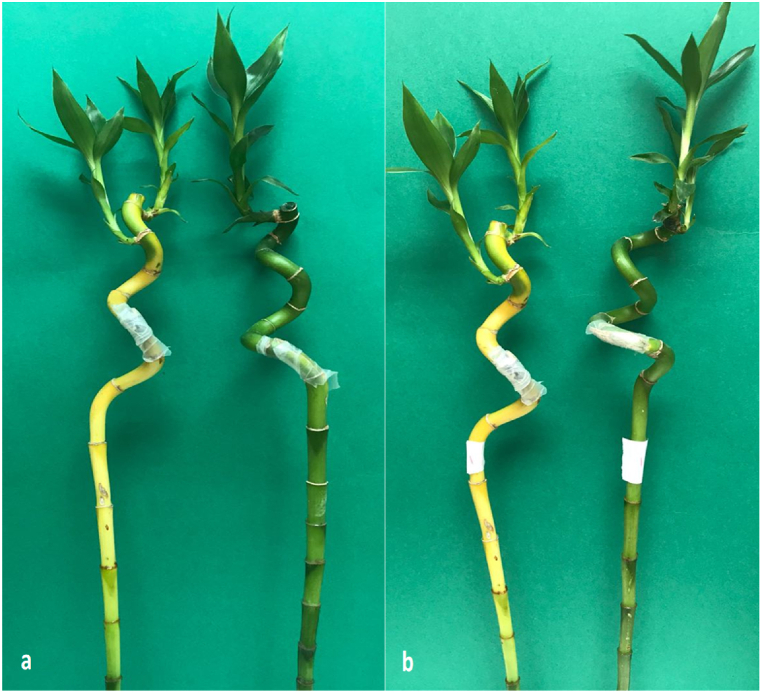
Preventive effect of thiophanate-methyl (a) and difenoconazole + azoxytrobin (b) against anthracnose of lucky bamboo plants (right) compared to un-treated control (left) under artificial inoculation with *C dracaenophilum* isolate 4. To achieve preventive effects, two months-old rooted cuttings of lucky bamboo plants were sprayed with a hand sprayer until runoff and kept in wide-mouthed, 2 L capacity glass jars containing 1 L water until inoculation. These jars were also amended with the same fungicide concentration (20 ppm). Control plants were similarly sprayed with sterile water and kept in separate glass jars to avoid contamination. Plants were inoculated by inserting a mycelia agar plug (5 mm in diameter) into a cut on the upper half of the stalk 24 h after fungicide spraying and kept at room temperature for 25 days. Photographs were taken 7 days after inoculation.

**Fig. 8:**
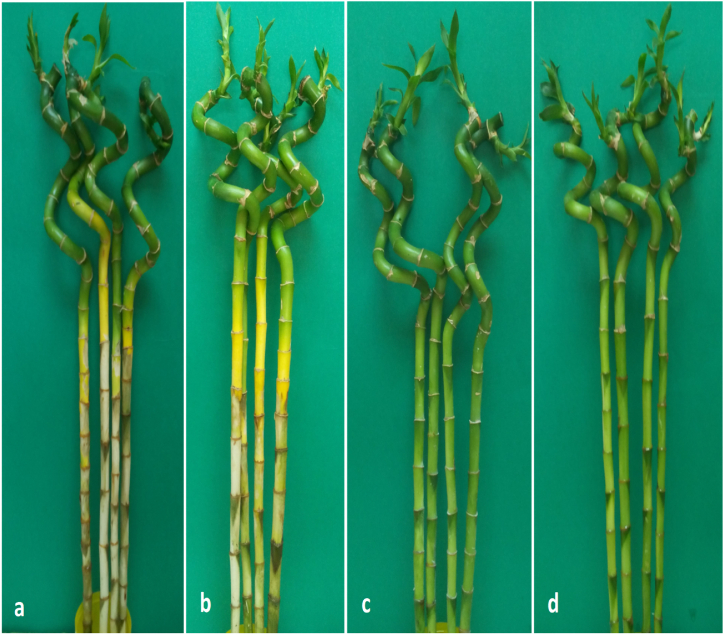
Phytotoxicity effect of Tebuconazole (a) and Flusllazole (b) and non-toxic effect of Thiophanate-methyl (c) and Difenoconazole + Azoxytrobin (d).

For preventive effects, the two fungicides thiophanate-methyl or difenoconazole + azoxytrobin were effective in protecting lucky bamboo plants from anthracnose infection. During the experiment’s evaluation phase, no indications of anthracnose appeared on non-inoculated control plants (25 days). Without fungicide, inoculated lucky bamboo plants developed more severe disease than plants treated with thiophanate-methyl or difenoconazole + azoxytrobin (Fig. 6). Inoculated lucky bamboo plants treated with difenoconazole + azoxytrobin had a 68.5% reduction in final disease severity compared to inoculated plants treated with thiophanate-methyl 53.3% or water 0.0% (Fig. 6, Fig. 7a,b). Because the fungicides tebuconazole and flusllazole caused phytotoxicity in lucky bamboo plants (Fig. 8a–d), the results of their preventive and curative effects are not shown.

### 3.5 Curative effect of thiophanate-methyl and difenoconazole + azoxytrobin

**Fig. 9:**
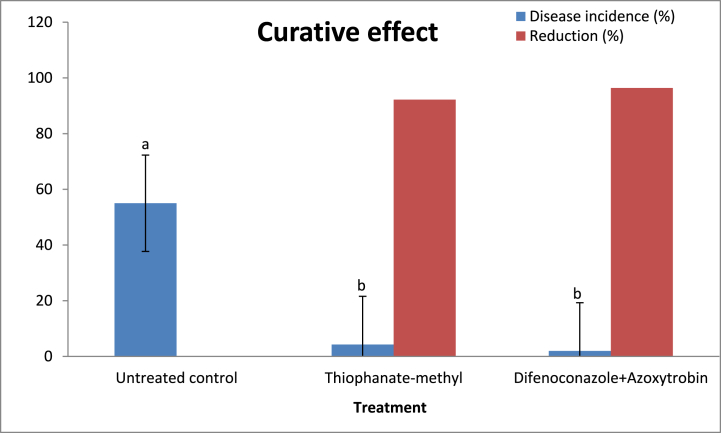
Curative effect of thiophanate-methyl and difenoconazole + azoxytrobin against anthracnose disease incidence (%) of non-inoculated lucky bamboo plants, 60 days after treatment. Values are means of ten replications. One week-old rooted cuttings of lucky bamboo plants were treated with each fungicide. At 60 days after treatments, the percent (%) of lucky bamboo plants with anthracnose symptoms was evaluated. According to Duncan’s multiple range tests, bars within each variable with the same letter show that the means and standard errors are not significantly different at P = 0.05. For analyses of variance, percentage data were converted using the arcsine square-root transformation; however, untransformed data are shown here.

**Fig. 10:**
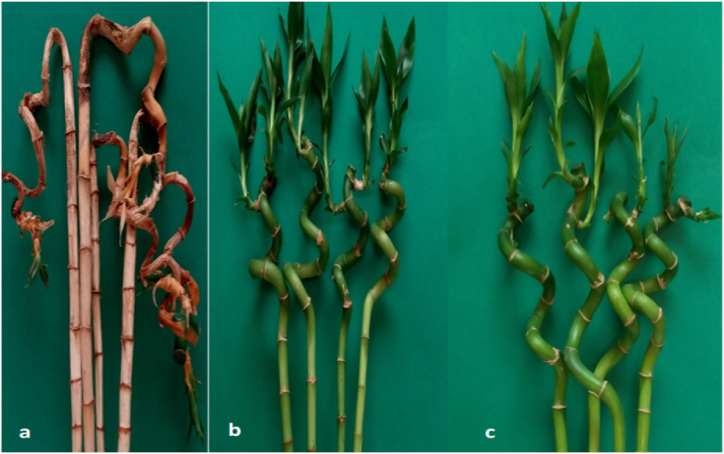
Curative effect of thiophanate-methyl (b) and difenoconazole + azoxytrobin (c) against anthracnose of lucky bamboo plants compared to un-treated control (a) under natural infection with *C. dracaenophilum*. For curative effects, one week-old rooted cuttings of lucky bamboo plants were sprayed with each fungicide but not inoculated and left in the same conditions for 2 months to see if disease would develop from asymptomatic plants and if the fungicides had a curative effect on *Colletotrichum* spp. Lucky bamboo plants were sprayed with each fungicide with a hand sprayer until runoff, then placed in wide-mouthed, 2 L glass jars containing 1 L water. These jars were also amended with the same fungicide concentration. The fungicide treatment was repeated at 25-day intervals, so each group received three treatments. Photographs were taken 60 days after the first application of the fungicide.

For curative effects, untreated plants that were left at room temperature for two months developed anthracnose symptoms (55.0% disease incidence) (Fig. 9). Overall, non-inoculated plants exposed to water had a higher number of diseased plants [55.0% disease incidence] than non-inoculated plants exposed to fungicides (Fig. 10a–c). Non-inoculated plants treated with thiophanate-methyl or difenoconazole + azoxytrobin have a low disease incidence of 4.3 and 2.0%, respectively, supportive the theory that these fungicides have a curative effect on *C. dracaenophilum* latent infection on lucky bamboo.

## 4 Discussion

Four *Colletotrichum* isolates were isolated from *D. sanderiana* plants with anthracnose symptoms in this study. The current study’s data show that all isolates were pathogenic to lucky bamboo and were re-isolated from inoculated plants, meeting the requirements for Koch’s postulates. Two *Colletotrichum* isolates from lucky bamboo were identified through sequencing as being *C. dracaenophilum*, whereas the remaining isolates belonged to the *C. gloeosporioides* species complex. These results are consistent with those obtained by Cannon et al. [28]. Both several *Colletotrichum* species infecting a single host and multiple *Colletotrichum* species infecting multiple hosts are frequent occurrences [[29], [30], [31]]. For the first time in Brazil, Macedo and Barreto [32] isolated *C. dracaenophilum* from *D. braunii* (lucky bamboo). *C. gloeosporioides* was identified as the causal pathogen of Pleomele (*D. reflexa* Lam.) anthracnose disease by Banerjee et al. [33]. According to Abdel-Rahman et al. [5], *C. gloeosporioides* was the most common of twelve different fungal pathogens identified from lucky bamboo (*D. sanderiana*) plants (26.93 and 26.34% for leaves and stems, respectively). In the current study, compared to the two isolates of the *C. gloeosporioides* species complex, *C. dracaenophilum* isolate 4 produced more severe disease on lucky bamboo plants and was isolated more frequently. These results have already been seen by Phoulivong et al., [34]. Thus, the *C. gloeosporioides* species complex member discovered on lucky bamboo in Egypt could have been present by chance when a susceptible ornamental plant was grown in the same greenhouse [34]. The greenhouse’s higher temperatures may have accelerated disease development. *Colletotrichum* species are the most common pathogens responsible for latent infections [35,36]. This is in line with the enlargement of lesions brought on by *C. lagenarium* in cucumber anthracnose and *C. truncatum* in lentil anthracnose [37,38]. The plants may have become more vulnerable as the temperature rose from 25 to 30 °C, or the *Colletotrichum* strain may have become more virulent at higher temperatures [9].

For preliminary screening and assessing the effectiveness of fungicides in treating a particular fungal pathogen, *in vitro* fungicide testing is essential. In this investigation, all the fungicides tested generally reduced *C. dracaenophilum* mycelial growth to some extent, and their inhibitory impact grew stronger with concentration. Thiophanate-methyl and difenoconazole + azoxytrobin were the most effective against *C. dracaenophilum*, completely inhibiting linear growth at ≥15 ppm. Tebuconazole and flusllazole were also effective, inhibiting *C. dracaenophilum* growth completely at ≥20 ppm. According to Yu et al. [39], azoxystrobin acts as an electron transport inhibitor by binding to the Qo center of cytochrome *b* (cyt *b*) and inhibited mycelial respiration within 12 h. Azoxystrobin inhibited mycelial growth and conidia germination in *C. capsici* and three other pathogens, according to Li-hua et al. [40]. Furthermore, an oxygen consumption test of mycelia revealed that azoxystrobin inhibited the respiration of all four fungi in the early stages. Thiophanate-methyl, a benzimidazole fungicide, on the other hand, inhibits the growth of fungal pathogens by interfering with microtubule assembly and selectively inhibits the proliferation of sensitive strains [41]. Chung et al. [42] discovered that *C. gloeosporioides* isolates were susceptible to three fungicides, namely iminoctadine-triacetate, thiophanate-methyl, and diethofencarb. On the other hand, all of the *C. acutatum* isolates were less sensitive. Difenoconazole suppressed *C. truncatum’s* mycelial growth, which causes chilli anthracnose, at concentrations of 10 ppm (79.44%) and 25 ppm (90%) according to Gopinath et al. [42]. According to Javaid et al. [43], all of the dithane or ridomil gold doses used significantly reduced the fungal biomass of *C. gleosporioides* Penz. Gawade et al. [44] evaluated the efficacy of five fungicides against *C. truncatum* and discovered that difenoconazole was one of the most effective, inhibiting it by 82.91% at 100 ppm. Difenoconazole, which is used to treat sapota fruit leaf blight (chikoo), was discovered by Patil et al. [45] to totally block the mycelial growth of *C. gloeosporioides*. Lima et al. [46] found that 0.5 ppm difenoconazole inhibited (by 60%) mycelial growth of *C. fructicola*, which causes mango anthracnose. According to Espinoza-Altamirano et al. [47], *C. acutatum* isolates were sensitive to azoxystrobin but moderately resistant to methyl thiophanate. *In vitro* tests of the fungicides performed by Nuraini and Latiffah [15] demonstrated that *C. fructicola*, *C. siamense*, *C. truncatum*, *C. scovillei*, and *C. fioriniae’s* mycelial growth was substantially reduced by the two systemic fungicides benomyl and difenoconazole. According to Chaudhuri et al. [11], difenoconazole with an EC_50_ value of 299.2/mL provided the best results against *C. fragrans* radial growth.

On lucky bamboo plants, the systemic fungicides thiophanate-methyl or difenoconazole + azoxytrobin were successful in preventing new *C. dracaenophilum* infection, curing latent infection, and preventing anthracnose development. Approximately 55.0% of non-inoculated lucky bamboo plants that were not treated with fungicide developed anthracnose 2 months after the fungicide experiment began, whereas only 4.3 and 2.0% of similar plants treated with thiophanate-methyl or difenoconazole + azoxytrobin developed anthracnose, respectively. This results are in accordance with those obtained by Daugovish et al. [48]. They reported that the fungicides thiophanate-methyl or difenoconazole + azoxytrobin were effective at preventing *C. acutatum* from growing on strawberry planting materials. According to MacKenzie et al. [49] azoxystrobin, pyraclostrobin, and thiophanate-methyl all reduced strawberry mortality due to *C. gloeosporioides* when applied two days before inoculation. According to Sharma et al. [9], 0.075 g a.i./L azoxystrobin significantly reduced anthracnose development on both latently infected and inoculated lucky bamboo plants. Jilková et al. [50] used azoxystrobin as well as six other fungicides on *C. acutatum* isolates. According to Boersma et al. [51], thiophanate-methyl had the greatest reduction in the severity of dry bean anthracnose disease caused by *C. lindemuthianum* and, as a result, greater seed yield, lower pick, and larger seed size compared to the inoculated control. Chaudhuri et al. [11] demonstrated that difenoconazole was more effective than blitox, mancozeb, and chlorothalonil in inhibiting the diseased lesion of *C. fragrans* on *Dracaena.*

## 5 Conclusion

Imported lucky bamboo plants are frequently infected with the fungal pathogen *C. dracaenophilum* or *C. gloeosporioides* species. *C. dracaenophilum* isolates were found to be more common and caused more severe disease on lucky bamboo plants, whereas *C. gloeosporioides* isolates were only mildly pathogenic. This study confirmed that *C. dracaenophilum* is responsible for anthracnose of lucky bamboo in Egypt. Six systemic fungicides were tested for their effects on *C. dracaenophilum in vitro* and *in vivo*. Tebuconazole and flusllazole were effective *in vitro* but cause phytotoxicity in bamboo plants, so their use should be deleted. The two systemic fungicides, thiophanate-methyl and difenoconazole + azoxytrobin, were found to be effective in preventing and curative anthracnose disease in lucky bamboo caused by the fungus *C. dracaenophilum*.

## Declarations

### Author contribution statement

Ibrahim E. Elshahawy, Osama M. Darwesh: Conceived and designed the experiments; Performed the experiments; Analyzed and interpreted the data; Contributed reagents, materials, analysis tools or data; Wrote the paper.

### Funding statement

This research did not receive any specific grant from funding agencies in the public, commercial, or not-for-profit sectors.

### Data availability statement

Data will be made available on request. The complete data generated and analyzed in the current study is available in the manuscript. The data on accession numbers are available in the following links; https://www.ncbi.nlm.nih.gov/nuccore/OP030719.1?report=GenBank (OP030719); https://www.ncbi.nlm.nih.gov/nuccore/OP030720.1?report=GenBank (OP030720); https://www.ncbi.nlm.nih.gov/nuccore/OP030721.1?report=GenBank (OP030721); https://www.ncbi.nlm.nih.gov/nuccore/OP030722.1?report=GenBank (OP030722).

## Declaration of interest’s statement

The authors declare no conflict of interest.

## Additional information

No additional information is available for this paper.
